# Engineered ligand‐based VEGFR antagonists with increased receptor binding affinity more effectively inhibit angiogenesis

**DOI:** 10.1002/btm2.10051

**Published:** 2017-02-17

**Authors:** Shiven Kapur, Adam P. Silverman, Anne Z. Ye, Niv Papo, Darren Jindal, Mark S. Blumenkranz, Jennifer R. Cochran

**Affiliations:** ^1^ Dept. of Bioengineering Stanford University Stanford CA 94303; ^2^ Dept. of Ophthalmology Byers Eye Institute, Stanford University Stanford CA 94303; ^3^ Dept. of Chemical Engineering Stanford University Stanford CA 94303; ^4^ Stanford Cancer Institute Stanford University Stanford CA 94303

## Abstract

Pathologic angiogenesis is mediated by the coordinated action of the vascular endothelial growth factor (VEGF)/vascular endothelial growth factor receptor 2 (VEGFR2) signaling axis, along with crosstalk contributed by other receptors, notably α_v_β_3_ integrin. We build on earlier work demonstrating that point mutations can be introduced into the homodimeric VEGF ligand to convert it into an antagonist through disruption of binding to one copy of VEGFR2. This inhibitor has limited potency, however, due to loss of avidity effects from bivalent VEGFR2 binding. Here, we used yeast surface display to engineer a variant with VEGFR2 binding affinity approximately 40‐fold higher than the parental antagonist, and 14‐fold higher than the natural bivalent VEGF ligand. Increased VEGFR2 binding affinity correlated with the ability to more effectively inhibit VEGF‐mediated signaling, both in vitro and in vivo, as measured using VEGFR2 phosphorylation and Matrigel implantation assays. High affinity mutations found in this variant were then incorporated into a dual‐specific antagonist that we previously designed to simultaneously bind to and inhibit VEGFR2 and α_v_β_3_ integrin. The resulting dual‐specific protein bound to human and murine endothelial cells with relative affinities of 120 ± 10 pM and 360 ± 50 pM, respectively, which is at least 30‐fold tighter than wild‐type VEGF (3.8 ± 0.5 nM). Finally, we demonstrated that this engineered high‐affinity dual‐specific protein could inhibit angiogenesis in a murine corneal neovascularization model. Taken together, these data indicate that protein engineering strategies can be combined to generate unique antiangiogenic candidates for further clinical development.

## 1 Introduction

Protein ligands and receptors have been used as the basis for a number of successful biotherapeutics. As examples, etanercept, an Fc‐fusion of tumor necrosis factor receptor 2, was approved for treatment of rheumatoid arthritis^1^; aflibercept (VEGF‐Trap), an Fc‐fusion of VEGFR1 and VEGFR2 extracellular domains, was approved for treatment of pathologic angiogenesis^2^, ^3^; and recombinant TRAIL (TNF‐related apoptosis‐inducing ligand) is under investigation for oncology applications.^4^ Despite these successes, natural ligands or receptors often lack required attributes of a potent therapeutic such as desired target affinity or specificity, or optimal functional activity. In these cases, proteins with altered properties can be generated via directed or combinatorial engineering methods.^5^ Examples include engineered ligands with altered receptor binding profiles,^6^ receptors engineered to possess ultrahigh affinity to their cognate ligand,^7^ engineered ligands with improved cell trafficking,^8^ or receptor agonists engineered to function as antagonists.^9^

VEGF and its principal receptor, VEGFR2, have generated interest for their central role in pathologic angiogenesis,^10^ particularly with respect to supporting the survival and growth of tumors or aberrant blood vessel formation in ocular disease. FDA‐approved agents that target and inhibit the VEGF/VEGFR2 signaling axis include the anti‐VEGF monoclonal antibody bevicuzimab (Avastin), and, more recently, ziv‐aflibercept/aflibercept (Zaltrap/Eylea). While the development of these agents underscores the clinical utility of VEGF/VEGFR2 inhibition, it has also highlighted several challenges, including acquired resistance to therapy and limited efficacy in certain disease states and patient subsets.^11^, ^12^ At the same time, a wealth of accumulated evidence has established that pathologic angiogenesis is mediated by the coordinated action of a number of other receptors, including platelet derived growth factor receptor, Tie receptor, and α_V_β_3_ integrin receptor.^13^, ^14^, ^15^ These findings have spurred the development of molecules with improved pharmacological properties, in particular, ones that can target a broader set of ligand–receptor interactions responsible for mediating pathologic angiogenesis.^11^, ^16^

**Figure 1:**
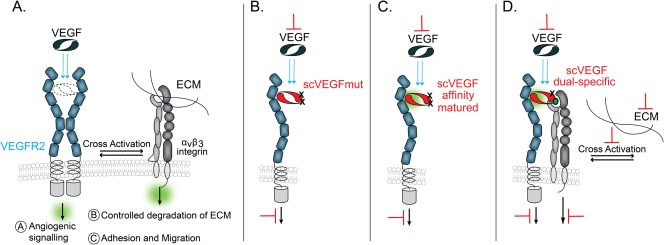
Design of VEGF‐derived antagonists. (a) Wild‐type VEGF (VEGF) binds to two copies of VEGFR2 and activates cell signaling. Residues from both chains of the VEGF homodimer interact with VEGFR2. (b) Single‐chain VEGF antagonist (scVEGFmut) has one VEGFR2 binding site mutated, preventing a second receptor molecule from binding, thereby blocking activation. (c) Single‐chain VEGF affinity‐matured antagonist contains mutations that enable it to bind more tightly to its target receptor and demonstrates more potent inhibition of VEGFR2 activation. (d) Single‐chain VEGF affinity‐matured dual‐specific antagonist (scVEGFdual‐specific) contains mutations that confer higher affinity binding to VEGFR2 and an engineered α_v_β_3_ integrin binding loop on the opposite pole of the molecule. We previously showed that dual‐specific proteins that bind both VEGFR2 and α_v_β_3_ integrin more potently inhibit angiogenic processes compared to mono‐specific proteins^27^

Previous studies have explored modifying the natural VEGF ligand to alter its function from a receptor agonist to that of a receptor antagonist. VEGF is a homodimeric protein that mediates endothelial cell growth, proliferation, and neovascularization through activation of the receptor tyrosine kinase VEGFR2 (Figure 1a).^17^ A VEGF homodimeric ligand binds to two molecules of VEGFR2, leading to receptor dimerization and autophosphorylation, and activation of intracellular signaling pathways, including PI3K, Src, Akt, and ERK.^18^ The concept of converting VEGF into an antagonist of VEGFR2 signaling was first explored by introduction of mutations that generated a monomeric form of the receptor,^19^ or that disrupted one pole of the VEGF/VEGFR2 binding interface, preventing dimerization and activation.^20^, ^21^ In another example, key amino acids involved in VEGFR2 recognition were mutated in VEGF (chain 1: E64R, chain 2: I46R), and the two subunits in the resulting heterodimer were connected via a 14‐amino acid linker, thereby creating a single‐chain VEGF (scVEGF) construct.^22^ Combination of both mutations on one pole of scVEGF abolished binding of one copy of VEGFR2; this scVEGF variant was found to inhibit the mitogenic effects of wild‐type VEGF protein on endothelial cells.^22^ In all of these examples, the monovalent VEGF ligand that resulted from these protein engineering efforts bound significantly weaker to VEGFR2 compared to the natural bivalent growth factor ligand due to loss of avidity effects, limiting the antagonistic potency of these inhibitors, and hence their clinical utility.

Motivated by the extensive biochemical cross‐talk between VEGFR2 and α_V_β_3_ integrin receptors that mediates pathologic angiogenesis^14^, ^16^, ^23^, ^24^, ^25^, ^26^ (Figure 1a), we previously described the conversion of scVEGF into a dual‐specific antagonist that targets both VEGFR and α_V_β_3_ integrin.^27^ In this work, we introduced four point mutations (chain 1 F17A, E64A; chain 2 I46A, I83A) on one VEGFR2 binding pole to create a variant (termed scVEGFmut) that retained VEGFR2 binding on the opposite pole, but was not capable of inducing receptor dimerization and activation (Figure 1b). Next, we introduced an engineered integrin‐binding loop into the mutated VEGF pole to generate a protein that simultaneously bound both VEGFR and α_V_β_3_ integrin. This dual‐specific protein was more a potent antagonist than the parent scVEGFmut, however, it has limited therapeutic potential as an inhibitor as its affinity is only comparable to that of the natural VEGF ligand. Here, we utilize protein engineering to address these limitations, first by generating a more potent ligand‐based antagonist by increasing VEGFR2 binding affinity (Figure 1c). Mutations identified in these screens that conferred increased VEGFR2 binding were then added to our previous VEGFR/α_V_β_3_ integrin antagonist (Figure 1d). The resulting high‐affinity dual‐specific protein binds to human endothelial cells with a relative affinity of 120 ± 10 pM, which is at least 30‐fold higher affinity than wild‐type VEGF (3.8 ± 0.5 nM). Additionally, the dual‐specific protein bound murine endothelial cells with a relative affinity of 360 ± 50 pM, and inhibited pathologic neovascularization in an in vivo model.

## 2 Results and discussion

### 2.1 Engineering a ligand‐based antagonist with high‐affinity binding to VEGFR2

**Figure 2:**
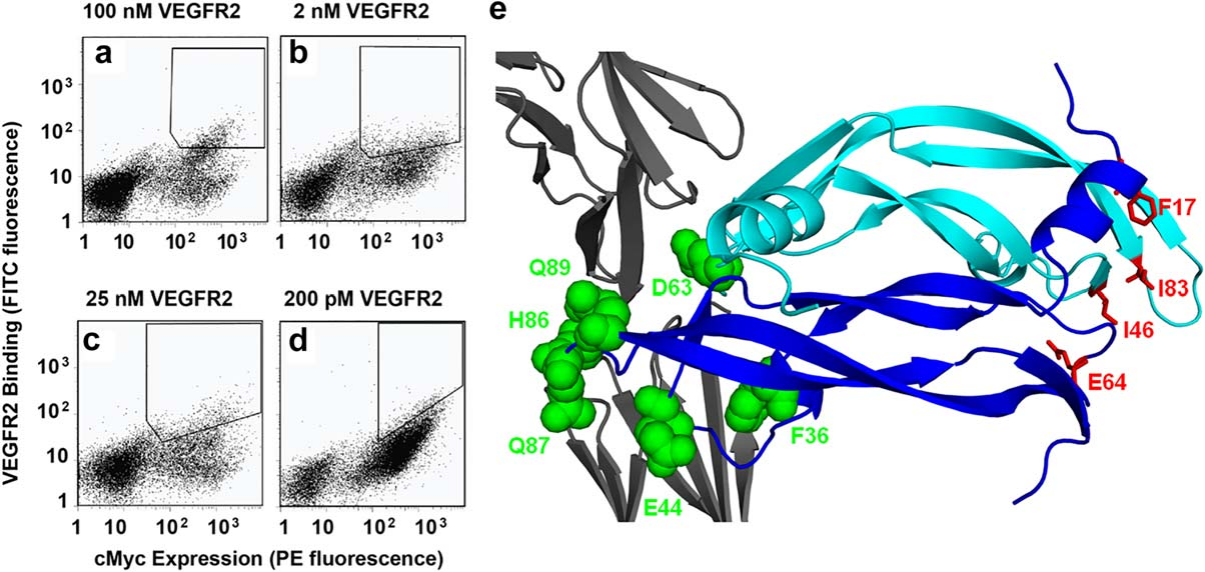
Library screening. FACS plots showing sorting of scVEGFmut error‐prone mutagenesis libraries. (a) Mutagenesis round 1, sort round 1, 100 nM VEGFR2‐Fc; (b) Sort round 6, 2 nM VEGFR2‐Fc; (c) Mutagenesis round 2, sort round 1, 25 nM VEGFR2‐Fc; (d) Sort round 6, 200 pM VEGFR2‐Fc; *x*‐axis indicates cMyc expression and *y*‐axis indicates receptor binding. Polygons indicate sort gates used to collect yeast cells. (e) Structure of wild‐type VEGF‐VEGFR2 complex (PDB: 3V2A) showing positions of the most common mutations (green spheres) selected from the scVEGF affinity maturation libraries. Chains 1 and 2 are shown in dark and light blue, respectively, and VEGFR2 is shown in gray. Mutations introduced into scVEGFmut to disrupt binding at the opposite pole of the protein are shown in red sticks

The mutations we previously introduced to generate the monovalent protein scVEGFmut (chain 1: F17A, E64A; chain 2: I46A, I83A) decreased its affinity for VEGFR2 due to loss of avidity effects (Figure 1b). Thus, we sought to identify variants with increased binding affinity by screening libraries of scVEGFmut proteins displayed on the yeast cell surface against soluble VEGFR2 extracellular domain (VEGFR2‐ECD). Libraries containing random mutations in scVEGFmut were created via error‐prone PCR using nucleotide analogs, with an average mutation frequency from ∼0.2 to ∼2%.^28^ DNA was electroporated into yeast by homologous recombination,^29^ resulting in a library of 0.5–2 × 10^7^ yeast transformants. Yeast displaying scVEGF variants were labeled with human VEGFR2‐ECD to measure receptor binding, and an anti‐cMyc antibody to quantify cell surface protein expression via a C‐terminal cMyc epitope tag. After treatment with fluorescently‐labeled secondary antibodies, the yeast showing the highest receptor binding relative to expression were selected by flow cytometric sorting and propagated, and the process was repeated for multiple rounds of screening (Figure 2).

After six rounds of library screening, in which sorting stringency was progressively increased by decreasing the concentration of VEGFR2‐ECD (100 nM in round 1 versus 2 nM in round 6; Figure 2a,b), the DNA encoding for enriched scVEGF variants from the last sort round was sequenced. Amino acid sequence analysis revealed neither consensus mutations nor convergence to a small number of variants. Hence, the DNA isolated from sort rounds 5 and 6 was recovered and used as a template for a second‐generation protein library created by error‐prone PCR. This library was enriched over 6 rounds of screening (Figure 2c,d), again using increased stringency at each progressive sort round. The final sort round for this library was performed using 200 pM VEGFR2‐ECD (Figure 2d), a concentration at which yeast‐displayed scVEGFmut and wild‐type VEGF (scVEGFwt) did not exhibit appreciable binding to the receptor.

### 2.2 Sequence analysis of isolated scVEGF variants revealed several consensus mutations

DNA sequencing from the final round of library screening highlighted a number of mutations common to multiple clones (Supporting Information Table 1). Significantly, each of these consensus mutations could be rationalized based on the existing crystal structure of the VEGF‐VEGFR2 complex and complementary alanine‐scanning mutagenesis studies at the same interface.^30^, ^31^ The consensus mutations were located either within or adjacent to the intact VEGF‐VEGFR2 binding interface^30^ (Figure 2e). Every isolated variant contained one or more of the three mutations, chain 1 H86Y, Q87R, and Q89H, representing altered size and electrostatic changes in the primary VEGF‐VEGFR2 binding loop.^30^, ^31^ In prior mutagenesis studies, mutation of residues 82–84 led to a several 100‐fold decrease in binding affinity, while the H86A mutation only led to an approximately twofold decrease; mutation of residues Q87 and Q89 was not reported.^31^ Chain 1 E44, which was mutated to Gly in several clones, lies in a separate loop, but also within the VEGF‐VEGFR2 binding interface. The E44A mutation led to a fivefold increase in apparent affinity of VEGF for VEGFR2,^31^ potentially explaining the occurrence of the similar E44G mutation in several clones. Chain 2 D63N, which was found in eight of the nine unique clones also lies within the VEGF‐VEGFR2 binding interface,^30^, ^31^ Another common mutation, chain 1 F36L (Figure 2e), is not directly at the VEGF‐VEGFR2 binding interface, but is close spatially^30^ and presumably stabilizes the receptor‐bound conformation.

### 2.3 Engineered proteins bind with higher affinity to cell surface‐expressed VEGFR2

**Figure 3:**
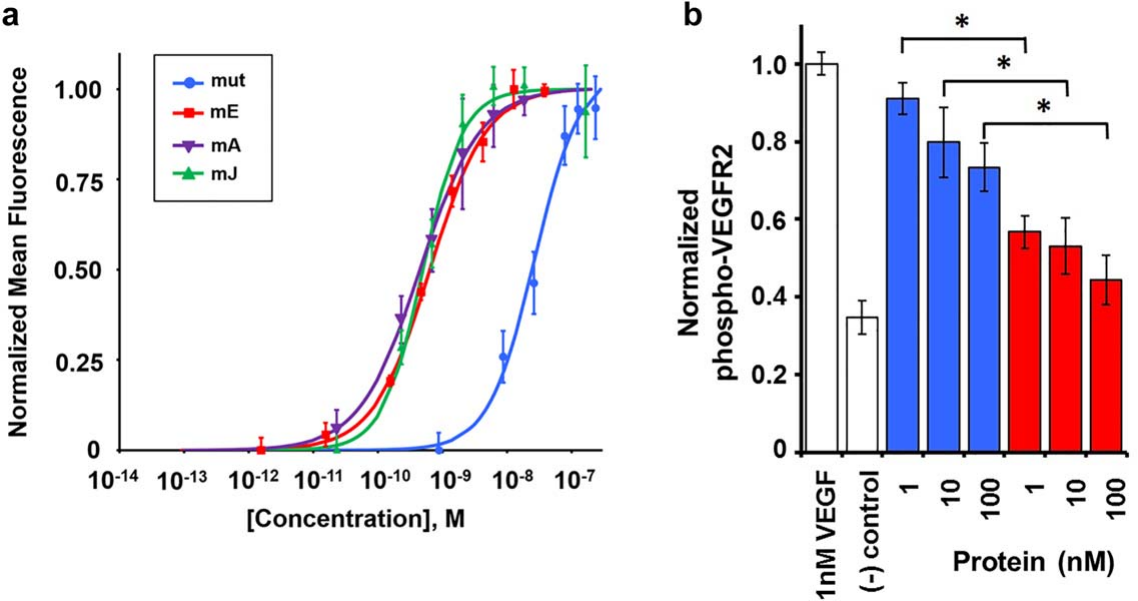
Characterization of scVEGF affinity‐matured variants. (a) Binding titrations on PAE/KDR cell line. Mean fluorescence values of variants were measured by flow cytometry using a fluorescently labeled antibody against a FLAG epitope tag. Data shown are the average of triplicate values and error bars represent standard deviations. (b) In vitro antagonistic activity of scVEGF variants. Effect of scVEGF variants on VEGF‐mediated tyrosine phosphorylation of VEGFR2 in HUVECs. Chemiluminescence read‐outs were quantified by densitometry. scVEGFmut (blue) or scVEGFmE (red). Data shown are the average of independent triplicate values, and error bars represent standard deviations. Compared to scVEGFmut, scVEGFmE was found to exhibit significantly improved inhibition of VEGFR2 phosphorylation at all three doses, by ANOVA with *p* < .0001

We selected variants mA, mE, and mJ for further study as they each contained many of the individual consensus mutations, and collectively, they constituted a set of proteins that allowed maximal sampling of mutations that were not common across clones (Supporting Information Table 2). These clones, along with scVEGFmut, were recombinantly expressed in *Pichia pastoris* (see Section 4 and Supporting Information Figure 1). We measured the binding affinity of the scVEGF variants against porcine aortic endothelial (PAE) cells that were stably transfected to express human VEGFR2 (PAE/KDR cells). The scVEGF variants mA, mE, and mJ bound to PAE/KDR cells with similar binding affinities (mE: 0.64 ± 0.01 nM; mA: 0.5 ± 0.1 nM; mJ: 0.50 ± 0.03 nM), approximately 40–55‐fold higher than scVEGFmut (27 ± 2 nM) (Figure 3a). scVEGFmE was chosen for subsequent in vitro and in vivo studies, as this variant contained the fewest number of mutations. Additionally, unlike mE, variants mA and mJ contained mutations on both poles of the ligand (Supporting Information Table 2); thus, we speculated that these mutations might be extraneous or could potentially confer undesirable bivalent VEGFR2 binding.

### 2.4 scVEGFmE exhibits high thermal and storage stability

We next examined the stability of scVEGFmE in two distinct, but complementary contexts: thermal stability and stability upon storage. Thermal melts were tracked by circular dichroism spectroscopy. We were unable to obtain complete thermal denaturation of scVEGFmE, even at 95°C, demonstrating the high stability of this protein (Supporting Information Figure 2). As a further test of stability, scVEGFmE was heated to 50°C, then after cooling its binding affinity to PAE‐KDR cells was determined and compared to protein that was not challenged thermally. Both proteins exhibited binding affinities that were indistinguishable (mE: 0.64 ± 0.01 nM, mE‐heated: 0.72 ± 0.01 nM) confirming the thermal stability of this protein (Supporting Information Figure 3). Next, we tested the stability with respect to long‐term storage by comparing the binding affinity of the same preparation of scVEGFmE tested 24 months apart and stored in phosphate buffered saline without any stabilizing agents at 2–8°C. Again, the binding affinities at the two‐time points were indistinguishable demonstrating remarkable stability on storage (data not shown).

### 2.5 Correlation of increased VEGFR2 affinity and inhibition of VEGF‐mediated activity

**Figure 4:**
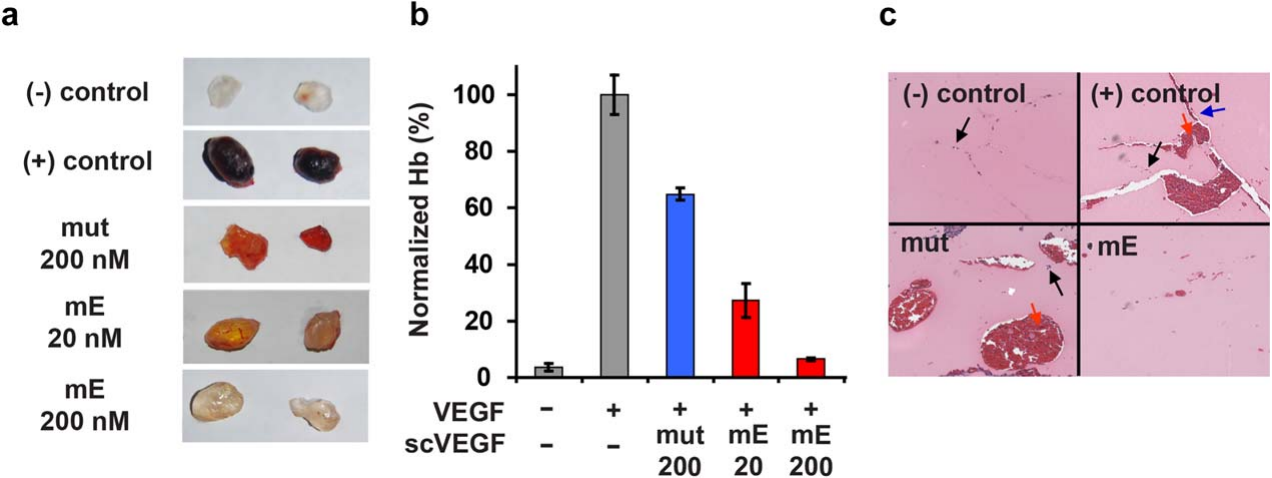
Inhibition of in vivo angiogenesis by scVEGF variants in Matrigel plugs implanted in C3H/HeN mice. Matrigel plugs contained PBS (− control), VEGF_165_/heparin (+ control), VEGF_165_/heparin + scVEGFmut (at 200 nM), or VEGF_165_/heparin + scVEGFmE (at 200 nm and 20 nM). (a) Plugs were removed from mice and photographed after 10 days. (b) Hemoglobin (Hb) content within Matrigel plugs was quantified and depicted as the % Hb compared to VEGF_165_/heparin (+ control). Reduction in Hb content for both scVEGFmE doses was determined to be statistically significant by ANOVA with *p* < .001, compared to the positive control as well as scVEGFmut. (c) H&E staining of 5‐μm Matrigel sections for blood vessel formation. Black arrows indicate nuclei, red arrows indicate red blood cells, and blue arrows indicate blood vessels

We measured the effects of scVEGFmE and scVEGFmut on VEGF‐mediated cell signaling in human umbilical vein endothelial cells (HUVECs). The engineered high affinity variant scVEGFmE more strongly inhibited VEGF_121_‐stimulated VEGFR2 phosphorylation compared to scVEGFmut (Figure 3b). Next, an in vivo Matrigel plug assay^32^ was performed to determine the ability of the scVEGF variants to inhibit angiogenesis induced by VEGF_165_ (see Section 4). Subcutaneous injection of Matrigel solutions into the flanks of C3H/HeN mice included positive control (VEGF_165_/heparin); negative control (no VEGF_165_); VEGF_165_/heparin/200 nM scVEGFmut; and VEGF_165_/heparin/200 or 20 nM scVEGFmE treated groups. Ten days later, the plugs from mice treated with positive control or with VEGF_165_/heparin/scVEGFmut were red in color (Figure 4a). In contrast, the plugs from mice treated with VEGF_165_/heparin/200 nM scVEGFmE were pale in color, similar to the plugs from the PBS‐treated negative control group. To obtain a quantitative readout of the degree of angiogenesis inhibition we measured the hemoglobin content (Figure 4b). Matrigel plugs from the 200 nM scVEGFmE treated group had 90% less hemoglobin relative to the positive control group. In comparison, scVEGFmut was only marginally effective at reducing hemoglobin content (*p* value <.001 for both scVEGFmE groups compared to scVEGFmut). Furthermore, while blood vessels and red blood cells were visualized in the positive control and the VEGF_165_/heparin/scVEGFmut groups by H&E staining of Matrigel plug sections, none or few red blood cells were observed in the 200 nM scVEGFmE treatment group (Figure 4c). These results demonstrate that the affinity matured variant scVEGFmE more potently inhibits VEGF‐mediated cell signaling and angiogenic processes compared to scVEGFmut.

### 2.6 scVEGFmE7I: A dual‐specific agent that binds VEGFR2 and α_v_β_3_ integrin with high affinity

Significant cross‐talk and synergy exists between VEGFR2 and α_v_β_3_ integrin in the context of pathologic angiogenesis.^14^, ^23^, ^24^ The coordinated signaling mediated by these two receptors is necessary for the angiogenic cascade which includes endothelial cell proliferation, controlled remodeling of the extra‐cellular matrix, and cell adhesion and migration^16^ (Figure 1a). As VEGFR2 and α_v_β_3_ integrin possess a reciprocally stimulatory relationship,^25^, ^26^ more potent antiangiogenic effects should be observed on blocking both receptors (Figure 1d). Indeed, in a previous study, co‐administration of mono‐specific inhibitors of VEGFR2 and α_v_β_3_ integrin showed more complete inhibition of angiogenesis in a mouse model compared to modest inhibition for single‐agent treated groups.^33^

Leveraging these biochemical insights, we previously engineered a first‐in‐class dual‐specific protein, scVEGF7I, which was comprised of scVEGFmut and an additional introduced epitope that enabled simultaneously binding to VEGFR2 and α_v_β_3_ integrin.^27^ In this prior study, scVEGF7I showed superior efficacy in inhibiting angiogenic processes relative to mono‐specific inhibitors of VEGFR2 and α_v_β_3_ integrin.^27^ However, the relatively modest binding affinity of this engineered dual‐specific antagonist for human endothelial cells (single digit nM) offers opportunities for further improvement. To achieve this goal we introduced the seven amino acid mutations identified in scVEGFmE into the scVEGF7I protein. The resulting dual‐specific protein, scVEGFmE7I, was expressed and purified in a similar manner as described above.

**Figure 5:**
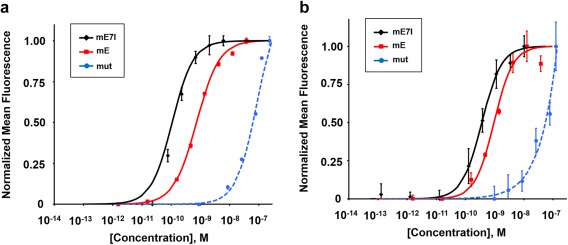
Binding titrations of scVEGF variants on mammalian cell lines. (a) HUVECs and (b) SVR cell line. Mean fluorescence values of variants were measured by flow cytometry using a fluorescently labeled antibody against a FLAG epitope tag. Due to the relatively weak binding of scVEGFmut to these two cell lines, we were unable to obtain saturation with this variant. Consequently, the curve (dotted line) is reported normalized to the fluorescence value corresponding to the highest tested concentration. Data shown are the average of triplicate values, and error bars represent standard deviations

We showed that scVEGFmE7I bound to HUVECs with an apparent affinity of 120 ± 10 pM, which is 30‐fold tighter than wild‐type VEGF (scVEGFwt) and at least 330‐fold tighter than the parental scVEGFmut antagonist (Figure 5a, Supporting Information Figure 4). In comparison, scVEGFmE bound to HUVECs with an apparent affinity of 750 ± 10 pM. As the affinity maturation screens were performed with human VEGFR2, we determined the apparent binding affinities of scVEGFmE and scVEGFmE7I to VEGFR2 expressed on mouse endothelial cells (SVEN 1 ras cells, SVR^35^) to facilitate preclinical testing in murine models. The extracellular domains of human and murine VEGFR2 share 85% sequence homology.^34^ scVEGFmE exhibited a similar binding affinity on SVR cells compared to HUVECs (apparent *K*_d_ = 950 ± 150 pM vs. 750 ± 10 pM, respectively). scVEGFmE7I bound to SVR cells with an apparent affinity of 360 ± 50 pM (Figure 5b). HUVEC and SVR cell lines have both been shown to endogenously express VEGFR2 and α_v_β_3_ integrin.^27^ However, the increase in relative binding affinity observed with scVEGFmE7I as compared to scVEGFmE is lower than what might be expected from a dual‐specific protein. One possible explanation is that avidity effects can be affected by receptor density or ineffective receptor clustering. Furthermore, there are significant challenges with measuring high affinity binding interactions to receptors expressed on the surface of mammalian cells, including inadequate time to reach equilibrium and ligand depletion effects;^36^ thus the reported values should be considered as relative estimates.

We next examined the thermal stability of scVEGFmE7I by circular dichroism spectroscopy. As before, we were unable to obtain complete thermal denaturation of scVEGFmE7I, even at 95°C (Supporting Information Figure 2), demonstrating the high stability of this protein that was comparable to the scVEGFmE.

### 2.7 Engineered high‐affinity dual‐specific antagonist inhibits angiogenesis in vivo

**Figure 6:**
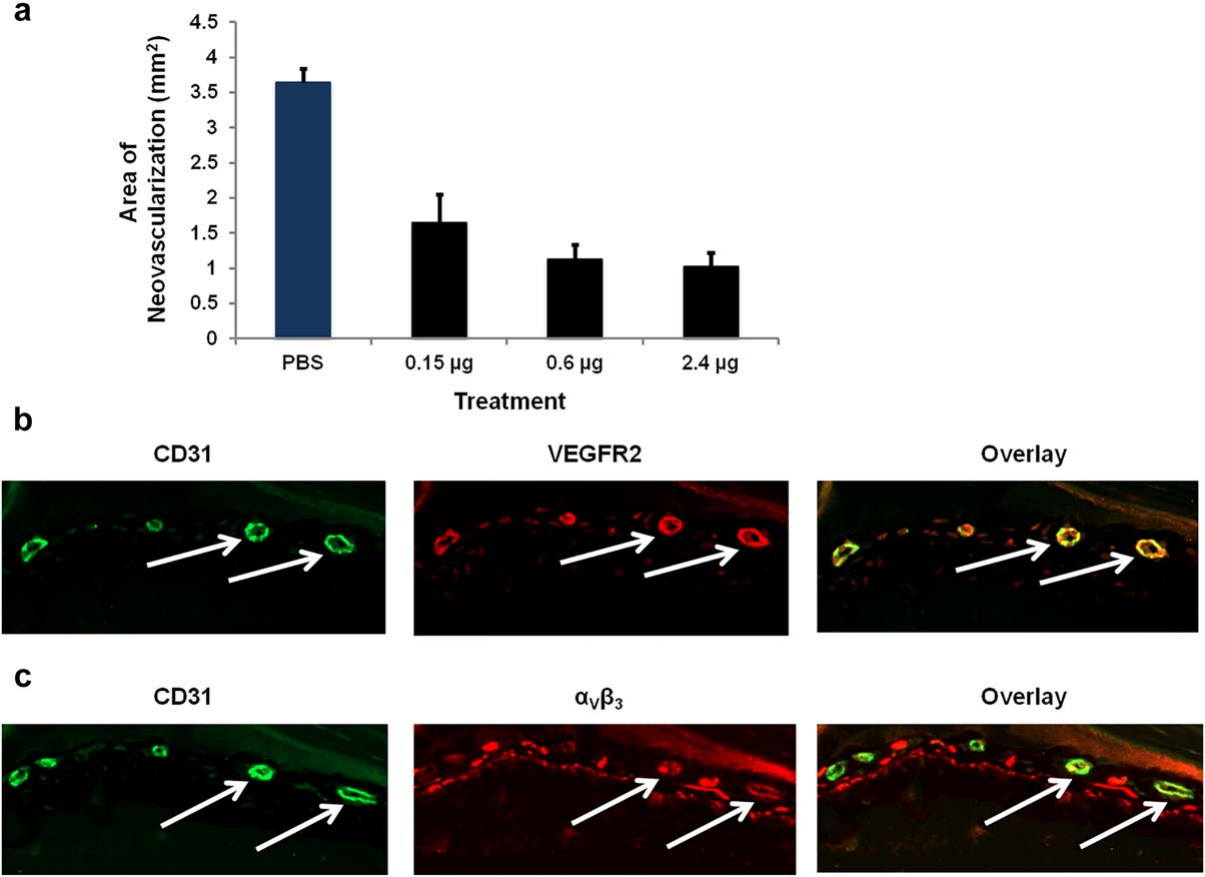
In vivo model for corneal neovascularization. (a) Area of neovascularization resulting from growth factor implanted pellet was measured for saline‐treated group (PBS) and the indicated three doses of scVEGFmE7I. Inhibition of neovascularization for all three doses was determined to be statistically significant by ANOVA with *p* < .001, compared to PBS‐treated control. (b,c) Immunofluorescent staining to visualize expression of VEGFR2 (b) and α_v_β_3_ integrin (c) on vasculature in tissue isolated from the PBS‐treated control. CD31 was used as a marker for vasculature (endothelial cells). White arrows indicate vasculature. For α_v_β_3_ integrin, staining of additional cell types present in the vicinity of vasculature is visible. It is noteworthy that this extra‐vascular staining is present only during angiogenesis as evidenced by its absence in the sham pellet control group (Supporting Information Figure 5D)

We next tested the ability of scVEGFmE7I to inhibit angiogenesis in an in vivo model of neovascularization. For these studies, we chose a mouse model for corneal neovascularization that has been previously used to evaluate the in vivo efficacy of angiogenesis inhibitors.^37^, ^38^, ^39^ The target tissue in this model expresses both VEGFR2 and α_v_β_3_ integrin receptor.^40^ For therapeutic delivery, a saline control (*n* = 3) or varying concentrations of mE7I (*n* = 5 for each concentration) were formulated within a pellet that is implanted into the cornea. A stimulatory growth factor was included in each pellet to promote angiogenesis. Six days after pellet implantation, the resulting neovascular area was quantified by imaging (Supporting Information Figure 5). At all doses tested, scVEGFmE7I implanted within each pellet was able to significantly inhibit neovascularization in this model relative to the saline control group (*p* < .001 for all treated groups) (Figure 6a). Immunofluorescent staining confirmed the expression of VEGFR2 and α_v_β_3_ integrin on vasculature in tissue isolated from the positive control (Figure 6b,c). Sham pellets containing saline and no growth factor showed that pellet implantation did not induce angiogenesis (Supporting Information Figure 5D). Further, the therapy did not appear to be acutely toxic at the doses administered as evidenced by measurement of body mass and daily external evaluation of the treated eye (Supporting Information Figure 5). As our previous work extensively compared the in vitro and in vivo efficacy of dual‐specific and mono‐specific VEGFR2 or α_v_β_3_ integrin targeting agents with different receptor binding affinities, we did not repeat these controls here, and simply sought to confirm that scVEGFmE7I could inhibit angiogenesis in a corneal neovascularization model as a next step for clinical translation.

## 3 Conclusions

This study highlights a strategy for engineering natural ligands as potential therapeutic leads for further clinical development. A caveat of this approach is that converting a bivalent, homodimeric ligand into a monomeric antagonist results in a significant decrease in receptor binding affinity due to loss of avidity effects, ultimately limiting therapeutic efficacy. The engineered ligand‐based antagonists scVEGFmE and scVEGFmE7I bind to human endothelial cells with significantly higher affinities compared to wild‐type VEGF. This affinity enhancement is important for the ability of such inhibitors to effectively outcompete natural stimulating ligands in a disease setting. While we used scVEGFmut for affinity maturation studies, we could have alternatively started with our first‐generation dual‐specific variant scVEGF7I to arrive at a dual‐specific variant with high affinity against integrin and VEGFR2. Multi‐specific protein therapeutics have generated great interest in drug development as they offer opportunities for: (a) improved therapeutic efficacy, (b) lower/less frequent dosing, and (c) lower risk of systemic exposure and off‐target effects.^41^, ^42^, ^43^ In particular, targeting VEGFR2 and α_V_β_3_ integrin using the high‐affinity dual‐specific angiogenesis inhibitor described in this work has the potential to reduce disease burden, and benefit patient subgroups that are either unresponsive or develop resistance to current therapies.

## 4 Materials and methods

### 4.1 Preparation of scVEGF constructs and libraries

The scVEGF constructs were prepared as described^27^ and cloned into the pCT yeast display plasmid. Library DNA containing random mutations was generated from scVEGFmut using error‐prone PCR and homologous recombination as described previously.^28^, ^44^ Briefly, a range of mutation frequencies (∼0.2–2%) was obtained using Taq polymerase (Invitrogen) and varying amounts of the nucleotide analogs 8‐oxo‐2′‐dGTP and 2′‐dPTP (TriLink Biotech) in separate PCR reactions consisting of 5 cycles (200 µM analogs), 10 cycles (2 or 20 µM analogs), or 20 cycles (2 µM analogs). PCR products were amplified in the absence of analogs, and a total of 10 μg of mutated cDNA insert and 1 μg restriction‐digested pCT backbone were transformed into EBY100 yeast by electroporation. To create a second‐generation library, plasmid DNA was extracted from yeast after rounds 5 and 6 of library sorting using a ZymoPrep kit (Zymo Research), and subjected to error‐prone PCR and yeast transformation as described above. In both cases, library sizes of ∼0.5–2 × 10^7^ transformants were obtained, as estimated by plating serial dilutions on selective media and colony counting.

### 4.2 Library screening by yeast surface display

Yeast‐displayed libraries were grown in selective media and induced for expression as previously described.^45^ Approximately 5–20 × 10^6^ yeast, depending on sort round, were labeled with VEGFR2 extracellular domain (Calbiochem EMD Chemicals) and a 1:200 dilution of chicken anti‐cMyc antibody (Invitrogen) in PBSA (phosphate buffered saline (PBS) containing 1 mg/ml bovine serum albumin [BSA]) for 2 hr at room temperature. After the incubation, cells were pelleted by centrifugation, the supernatant aspirated, and then the cells were resuspended in ice‐cold PBSA containing a 1:25 dilution of fluorescein‐conjugated anti‐VEGFR2 antibody (R&D Systems) and 1 mg/ml of Alexa Fluor 555‐conjugated anti‐chicken IgY antibody (Invitrogen). After 20 min of incubation with the antibody on ice, yeast were washed in PBSA and sorted using a Becton Dickinson FACSVantage SE instrument (Stanford FACS Facility) and CellQuest software. In each sort, ∼1–2% of yeast were collected, and after sort 1, the number of yeast analyzed was at least 10‐fold excess of the remaining library diversity. Concentrations of VEGFR2 used in each sort round were as follows: Library 1: sort 1–100 nM, sort 2–50 nM, sort 3–50 nM, sort 4–25 nM, sort 5–5 nM, sort 6–2 nM; Library 2: sort 1–25 nM, sort 2–5 nM, sort 3–2 nM, sort 4–1 nM, sort 5–500 pM, sort 6–200 pM. Plasmid DNA was extracted from yeast using a ZymoPrep kit (Zymo Research) and transformed into *Escherichia coli* XL‐1 Blue supercompetent cells (Stratagene). Individual clones were miniprepped using a Qiagen kit, and the eluted DNA was submitted for sequencing (MCLabs, S. San Francisco, CA).

### 4.3 Recombinant protein production and characterization

Protein production was performed using the Multi‐Copy *Pichia* Expression Kit (Invitrogen), using the pPIC9K plasmid (Invitrogen) and the *P. pastoris* GS115 yeast strain (Invitrogen). An N‐terminal FLAG epitope tag and a C‐terminal hexahistidine tag were included as handles for cell binding studies and protein purification, respectively. Endo H_f_ (New England Biolabs) was used to remove N‐linked glycosylation,^22^ and proteins were purified by metal chelating chromatography and size exclusion chromatography. Purified proteins were analyzed by SDS‐PAGE (NuPAGE 4–12% Bis‐Tris, Invitrogen) under nonreducing conditions (Supporting Information Figure 1). The purified proteins appeared as a single peak on analytical size exclusion FPLC and did not revert to multimers. Protein concentrations were determined by Bradford protein assay and UV‐Vis absorbance at 280 nm (ɛ_280_= 13,760 M.^−1^ cm^−1^ for scVEGFwt and scVEGFmut, and ɛ_280_= 15,040 M^−1^ cm^−1^ for scVEGFmA, 13,760 M^−1^ cm^−1^ for scVEGFmE, 15,040 M^−1^ cm^−1^ for scVEGFmJ, and 12,480 M^−1^ cm^−1^ for scVEGFmE7I). Purification yields for all proteins were 4–10 mg/L of culture. Proteins were filter‐sterilized before use in cell assays and mouse models, and were stored at 4°C.

For the scVEGFmE7I construct tested in the corneal neovascularization study, we incorporated two modifications. First, the original 14 amino acid linker connecting the two VEGF chains in the scVEGF scaffold (GSTSGSGKSSEGKG)^22^ was replaced by a longer linker comprised of (G_4_S)_4_. This replacement was guided by the insight that the maximum distance that can be bridged by the original linker (∼40 Å) is very close to the distance between the C‐terminus of monomer A and the N‐terminus of monomer B (∼38 Å) of the homodimeric VEGF.^46^ Second, we removed the N‐terminal FLAG epitope tag to eliminate potential in vivo artifacts originating from the inclusion of this sequence.

### 4.4 Cell binding assays

PAE and PAE/KDR cells were grown in F‐12 (Ham's) Nutrient Media (Gibco) with 10% fetal bovine serum (FBS) and 1% penicillin/streptomycin. HUVECs were grown in full EGM‐2 media (Lonza) containing 2% FBS and growth factor supplements. For all assays with HUVECs, low passage numbers (less than 8) were used, and cells were serum‐starved in basal media (EGM‐2 media without FBS and growth factor supplements) for 10–16 hr prior to being assayed. SVR cells were grown in Dulbecco's Modified Eagle Medium (ATCC) containing 10% FBS and 1% penicillin/streptomycin. Cells grown on T‐75 flasks were split at ∼70–80% confluence using cell dissociation buffer (Gibco). For cell binding assays, 50,000 cells were used per condition. Cells were suspended in integrin binding buffer (IBB, 0.1 ml volume, 20 mM Tris pH 7.5, 100 mM NaCl, 1 mM MgCl_2_, 1 mM MnCl_2_, 2 mM CaCl_2_, and 1 mg/ml BSA) and added to 1.8 ml of protein solution at the appropriate concentration, precooled to 4°C; these conditions were necessary and sufficient to avoid ligand depletion at all ligand concentrations tested. All subsequent steps were performed at 4°C. The cells were incubated with protein with gentle agitation to prevent settling for 4–6 hr, then pelleted at 1,340*g* for 5 min before the supernatant was aspirated. The cells were washed with PBSA (1 ml) and resuspended in 20 µl of PBSA containing a 1:50 dilution of phycoerythrin‐conjugated anti‐FLAG antibody (Prozyme). After 30 min of incubation with antibody on ice, the cells were resuspended in PBSA (1 ml), pelleted by centrifugation and the supernatant was aspirated. The cells were kept as pellets on ice and analyzed by flow cytometry immediately. Mean cell fluorescence for each protein concentration was calculated using FlowJo (Treestar, Inc.) then plotted versus log concentration. The data were fit to the following sigmoidal equation to calculate dissociation constants using KaleidaGraph (Synergy Software).

$$y\;=\;m_{1}+\frac{m_{2}-m_{1}}{1+{\left(\frac{x}{m_{3}}\right)}^{m_{4}}}$$

Error is reported as the standard deviation of triplicate measurements. None of the affinity‐matured scVEGF variants (mA, mE, or mJ) bound to the parental untransfected PAE cells up to 500 nM.

### 4.5 Circular dichroism spectroscopy

Circular dichroism spectroscopy was used to track thermal denaturation of scVEGF‐based proteins. Data was collected on a JASCO J‐815 instrument (JASCO, Easton, MD) equipped with a Peltier controller in a 0.1 cm cuvette from 20 to 95°C at 228 nm and an averaging time of 1 s.

### 4.6 VEGFR2 phosphorylation assays

VEGFR2 phosphorylation assays were carried out following a previously described protocol,^47^ with suitable modifications as described below. Briefly, subconfluent HUVECs were cultured in growth factor‐ and serum‐depleted EBM‐2 medium for 20 hr at 37°C/5% CO2 prior to experimentation. After pretreatment with 1 mM sodium orthovanadate (Na_3_VO_4_, Sigma) for 20 min, cells were co‐incubated with 1 nM VEGF_121_ (R&D systems) and different concentrations of scVEGF proteins for 10 min at 37°C. Cells were washed in PBS with 1 mM Na_3_VO_4_ and lysed for 2 hr in ice‐cold 1% Triton X‐100 lysis buffer (20 mM Tris pH 7.4, 150 mM NaCl, 1% TritonX‐100, 1× APC, 1× AEBSF [R&D systems], 1 mM Na_3_VO_4_, 1× complete protease inhibitor tablet [Roche]). Lysates were clarified by centrifugation (13,000 rpm for 10 min at 4°C), and protein concentrations were measured using a BCA protein assay kit (Thermo scientific). Equivalent amounts of protein from each lysate were subjected to SDS‐PAGE (NuPAGE 4–12% Bis‐Tris) and transferred to nitrocellulose (Invitrogen) for Western blot analysis. Blots were blocked in 5% milk, 20 mm Tris‐HCl pH 7.4, 150 mm NaCl, 0.3% Tween 20, and probed with a phospho‐specific rabbit polyclonal antibody (1:1,000 dilution; Y951‐VEGFR2, Abcam) overnight at 4°C. Immunoreactive bands were visualized using an HRP‐conjugated anti‐rabbit secondary antibody (1:2,000 dilution; Santa Cruz Biotechnology) and chemiluminescence (ECL plus, Amersham), and were quantified on a BioRad Chemidoc instrument. Blots were stripped and re‐probed with a rabbit polyclonal VEGFR2 antibody (Abcam) to determine the total amount of VEGFR2 for each sample. Unstimulated cells and cells stimulated with 1 nM VEGF_121_ were used as negative and positive controls, respectively. The intensities of the phospho‐VEGFR2 bands were adjusted for total VEGFR2 expression for each sample, and were normalized against the VEGF_121_‐only stimulated positive control. Data are presented as average values, and error bars represent the standard deviation of measurements performed in triplicate.

### 4.7 In vivo matrigel angiogenesis assay

Animal procedures were performed according to a protocol approved by the Institutional Animal Care and Use Committee. Anesthetized C3H/HeN mice (*n* = 3) were injected subcutaneously with a liquid Matrigel‐PBS mixture (650 µl/injection) as described previously.^48^ Matrigel contained human VEGF_165_ (20 nM, R&D systems) and 42 Units of heparin (Hospira) to stimulate angiogenesis, and scVEGFmut (at 200 nM) or scVEGFmE (at 200 or 20 nM). VEGF_165_ was used for stimulation, as this variant contains a heparin binding domain that aids in keeping the growth factor localized to the plug. Matrigel plus PBS only or 20 nM human VEGF_165_/heparin served as negative and positive controls, respectively. After 10 days, Matrigel plugs were removed and photographed, and hemoglobin content was determined according to an established method, with suitable modifications as described below.^49^ Briefly, dissected Matrigel plugs in PBS were treated with a Polytron homogenizer. Matrigel homogenate (15 μl) was added to 90% glacial acetic acid (135 μl) and incubated for 20 min. The samples were clarified by centrifugation (5,000*g*, 5 min), and supernatant (10 μl) was added to 3,3′,5,5′‐tetramethylbenzidine (140 μl, 5 mg/ml, Sigma) in 90% glacial acetic acid in a microtiter plate. Hydrogen peroxide (150 μl, 0.3%, Sigma) was added to each well, and the absorbance was measured at 600 nm. Relative hemoglobin content was calculated based on a hemoglobin standard curve (Sigma), and was normalized to the weight of the Matrigel plug. Percentage of hemoglobin reported for a given plug is normalized to the Matrigel plug containing 20 nM human VEGF_165_/heparin (+ control). Error bars represent the standard deviation of measurements performed on at least three Matrigel plugs. For histological evaluation, excised Matrigel plugs were fixed in 4% buffered formaldehyde, embedded in paraffin, and 5 μm sections were stained with hematoxylin and eosin (H&E).

### 4.8 Mouse model for corneal neovascularization

The in vivo corneal neovascularization model was performed as described previously.^37^, ^38^ All animal studies were performed according to a protocol approved by the Institutional Animal Care and Use Committee. Female C57BL/6 mice (C57BL/6NCrl; Charles River) were six weeks old and had a body weight range of 14.6–19.1 g on day 1 of the study. A batch of 100 pellets for use in the positive control group was prepared as follows. A vial of 10 μg basic fibroblast growth factor (bFGF; Invitrogen) was combined with 10 μl sterile PBS. Carafate (4 mg) was then added and the solution was briefly vortexed. A 12–15% Hydron solution (polyhydroxyethylmethacrylate, a slow release polymer) was added and mixed. Once a uniform solution was obtained, the solution was spread evenly onto a grid evenly using a cell scraper. Pellets were allowed to dry for 2–3 min, then ejected from the grid into a sterile petri dish and stored at 4°C. Pellets containing scVEGFmE7I were prepared identically except the appropriate concentration of engineered protein was used in place of saline. Sham pellets were prepared identically except that bFGF was omitted. bFGF was used as the angiogenic inducer as it has been shown to induce VEGF expression in this and other models.^50^, ^51^ Furthermore, bFGF induces the formation of relatively organized vasculature, assisting with quantification of neovascularization in this model.^52^

Mice were anesthetized then placed on a heating pads. Under a dissecting scope, a 2‐mm intrastromal linear keratotomy was made parallel to the lateral rectus muscle with a surgical blade. The incision was opened using forceps and an intrastromal pocket was made toward the lateral canthus using a modified corneal knife. A single pellet (PBS control or scVEGFmE7I with indicated dose or sham) was inserted and tunneled the remaining distance to within 1 mm of the temporal limbus. The pocket flap was closed allowing as little air to remain as possible and topical sterile saline was applied to the incision, followed by an application of nonhydrocortisone topical antibiotic. The pellet placement was within 0.95–1.05 mm of the limbus for all mice. The surgical site for all mice showed optimum healing with no deformities at incision site and the entire pellet packed and present at the end of the micropocket.

Positive control group (*n* = 3) received a pellet with 100 ng bFGF only. Groups treated with scVEGFmE7I (*n* = 5) received a pellet with 100 ng bFGF and the indicated dose of scVEGFmE7I (0.15 μg or 0.6 μg or 2.4 μg). Sham surgery control group (*n* = 3) did not contain bFGF or agent. Animals were weighed daily from day 1 to day 6. The mice were observed frequently for overt signs of any adverse, treatment‐related side effects, and clinical signs of toxicity (redness, inflammation, or discharge from the eye). No adverse effects were noted for any group. Animals were anesthetized and evaluated under a slit lamp microscope (Nikon FS3V). From a directly “head‐on” posture to the eye of the mouse, the contiguous circumferential zone of neovascularization (1 clock hour (CH) = 30°) was measured. The mouse was rotated approximately 30° in a manner to bring the limbal vascular plexus closer to view. The average vessel length (VL, in mm) was measured with a linear reticule through the slit lamp from limbal vessels toward the pellet. Neovascular area was calculated using the formula for half an ellipse:

$$\text{Area}\;({\text{mm}}^{2})\;=\;1/2\;\left(3.14\right)\;\times \;\text{VL}\;\times \;\text{CH}\;\times \;0.4.$$

Prism (GraphPad) was used for all statistical analyses. A one‐way ANOVA with Dunnett's multiple comparison test was employed to assess the significance of the difference between the mean areas of neovascularization between scVEGFmE7I treated groups and the positive control group (PBS).

For immunohistochemical staining, mouse eye sections were deparaffinized in xylene and rehydrated through a graded alcohol series to water. The slides were briefly rinsed, then subjected to heat mediated antigen retrieval in 10 mM sodium citrate buffer. Slides were washed in PBS, then incubated in 10% normal goat serum (Abcam) in PBS with 1% BSA for 3 hr at room temperature for blocking. Each section was then incubated for 12 hr at 4°C with a cocktail of two antibodies raised in differing species to achieve staining overlays. Antibodies for CD31 (Dianova), VEGFR2 (Abcam), and β_3_ integrin (Abcam) were used. Isotype control antibodies were purchased from Cell Signaling. PBS with 1% BSA was used for all antibody dilutions. The slides were then washed in PBS, and incubated for 1 hr at room temperature in Alexa Fluor 488 and Alexa Fluor 594 conjugated antibodies raised in goat against rat and rabbit, respectively (Life Technologies Molecular Probes, Carlsbad, CA). Slides were then washed in PBS, and mounted with 4′‐6‐diamidino‐2‐phenylindole‐containing Vectashield mounting media (VectorLabs). Fluorescence images were captured using a 10× Plan Apochromat objective on an AxioImager Z1 Epifluorescence Microscope (Carl Zeiss) with appropriate filter sets at the Stanford University School of Engineering Shriram Center Cell Sciences Imaging Facility. Exposure times for each antigen were constant across samples. All images of an antigen received analogous linear brightness and contrast adjustments using Zen Blue software (Carl Zeiss).

## Supporting information

Additional Supporting Information may be found online in the supporting information tab for this article.

Supporting Information
