# Supplementary material for: Engineered ligand‐based VEGFR antagonists with increased receptor binding affinity more effectively inhibit angiogenesis
Source: Bioeng Transl Med. 2017 Feb 17;2(1):81–91. doi: 10.1002/btm2.10051 (PMC5412928; doi:10.1002/btm2.10051)
Supplement: Supplementary file 1 — Supporting Information [file BTM2-2-081-s001.docx]

Engineered ligand-based VEGFR antagonists with increased receptor binding affinity more effectively inhibit angiogenesis

Shiven Kapur^1^, Adam P. Silverman^1^, Anne Z. Ye^1^, Niv Papo^1^, Darren Jindal^1^, Mark S. Blumenkranz^4^ and Jennifer R. Cochran^1,2,3,*^

Department of Bioengineering^1^, Department of Chemical Engineering^2^, Stanford Cancer Institute^3^, Stanford University, Stanford, California, 94305

Department of Ophthalmology^4^, Byers Eye Institute, Stanford University, Stanford California, 94305

* *Corresponding author*: Jennifer R. Cochran, 443 Via Ortega, Room 356, Shriram Center for Bioengineering and Chemical Engineering, Stanford University, Stanford, CA 94305-4125, USA. Email: jennifer.cochran@stanford.edu


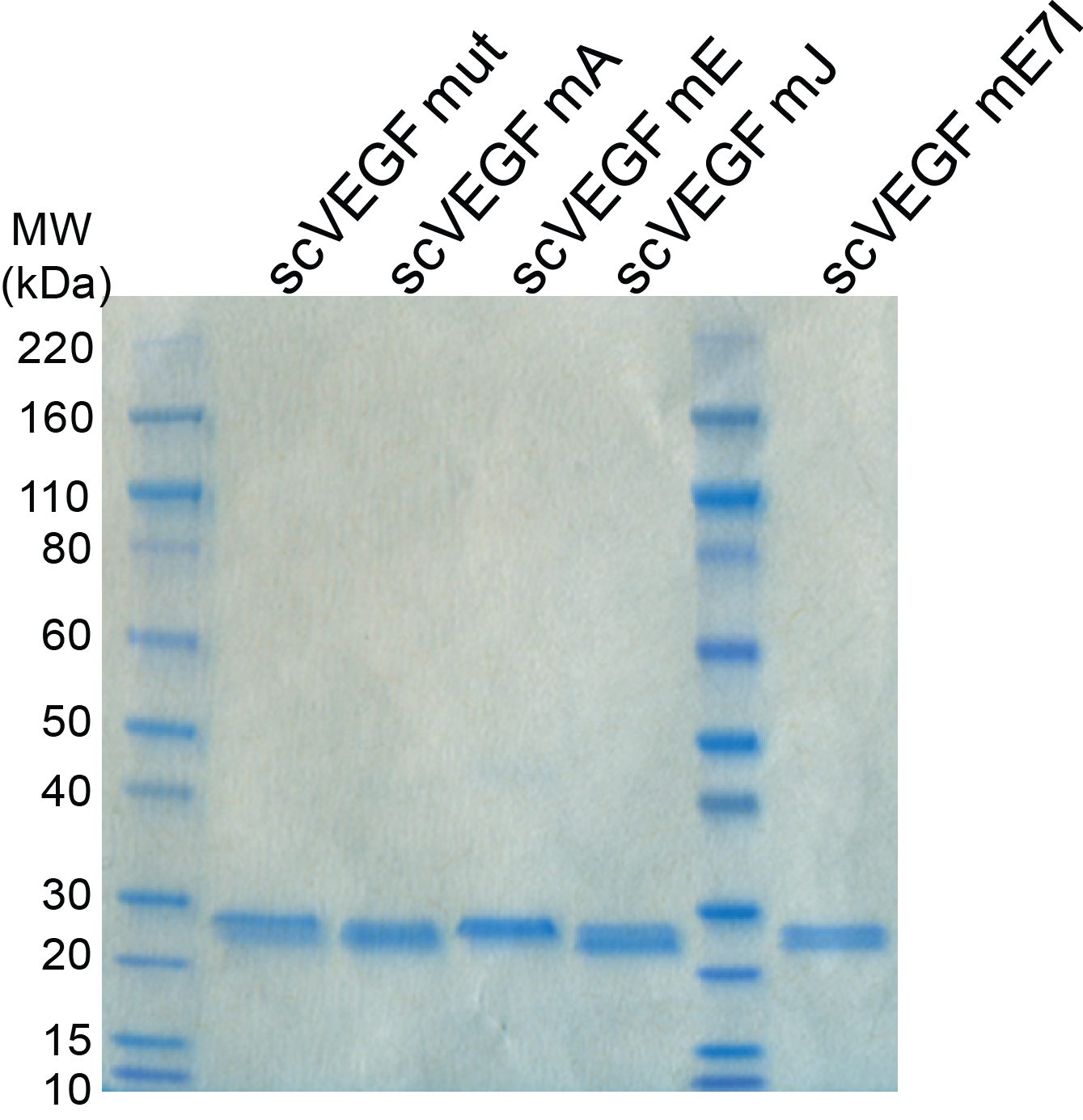


**Supporting Information Figure 1.** Analysis of purified scVEGF variants. SDS-PAGE analysis on a 4-12% polyacrylamide gel under non-reducing conditions. Bands were visualized by staining with Coomassie Blue. The lane headings correspond to either molecular weight markers or the indicated purified scVEGF proteins post-size exclusion chromatography. The purified proteins appeared as a single peak on analytical size exclusion FPLC and did not revert to multimers upon storage in PBS at 2-8 °C for up to 24 months as determined by SDS-PAGE.


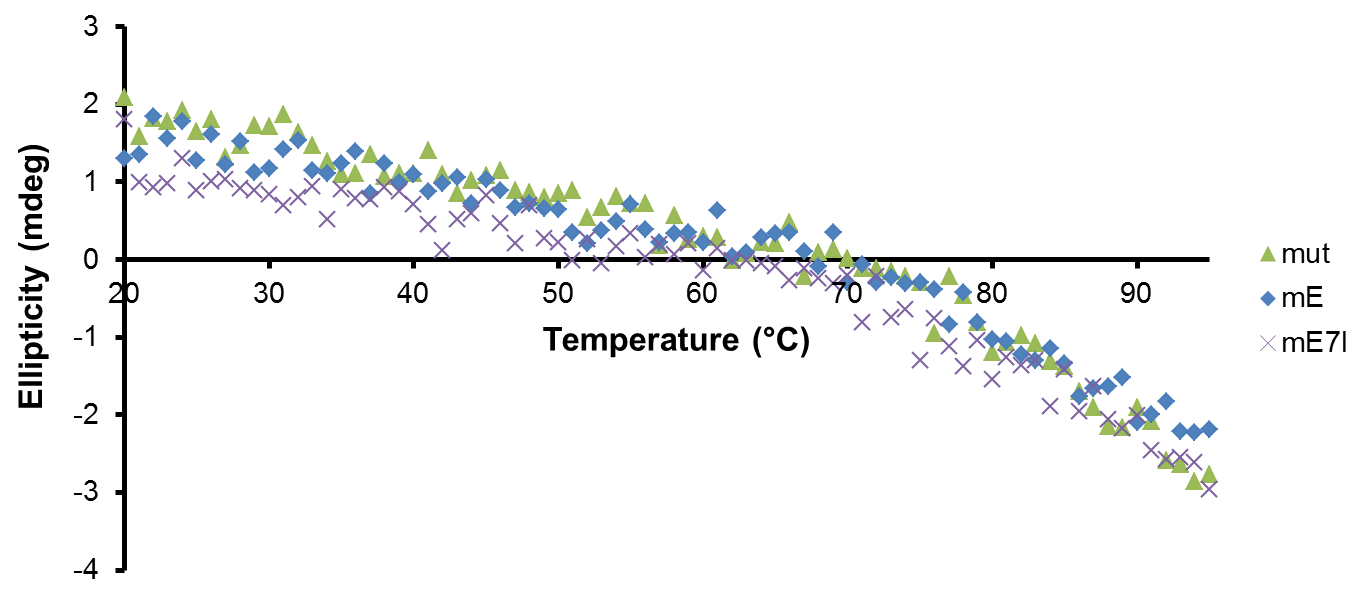


**Supporting Information Figure 2.** Thermal melts tracked by circular dichroism spectroscopy. Spectra for scVEGFmut, scVGEFmE, and scVEGFmE7I appear similar. We were unable to obtain complete thermal denaturation, even at 95 °C, demonstrating the high stability of these proteins.


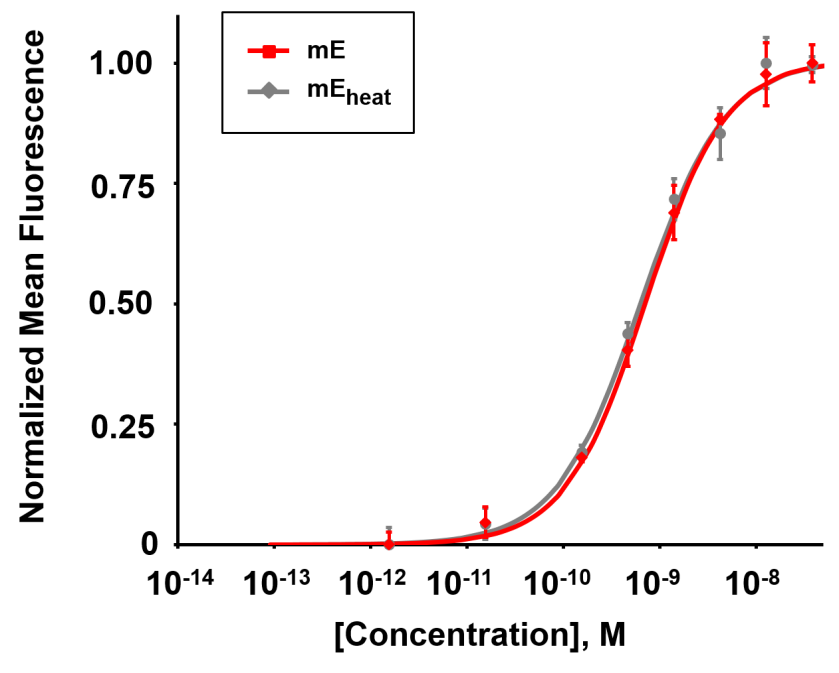


**Supporting Information Figure 3.** Functional assay to measure thermal stability. Binding assay with scVEGFmE and scVEGFmE-heated (heated to 50 °C) on PAE-KDR cells. The two proteins exhibited comparable binding affinities (640±10 pM and 720±10 pM for scVEGFmE and scVEGFmE-heated, respectively).


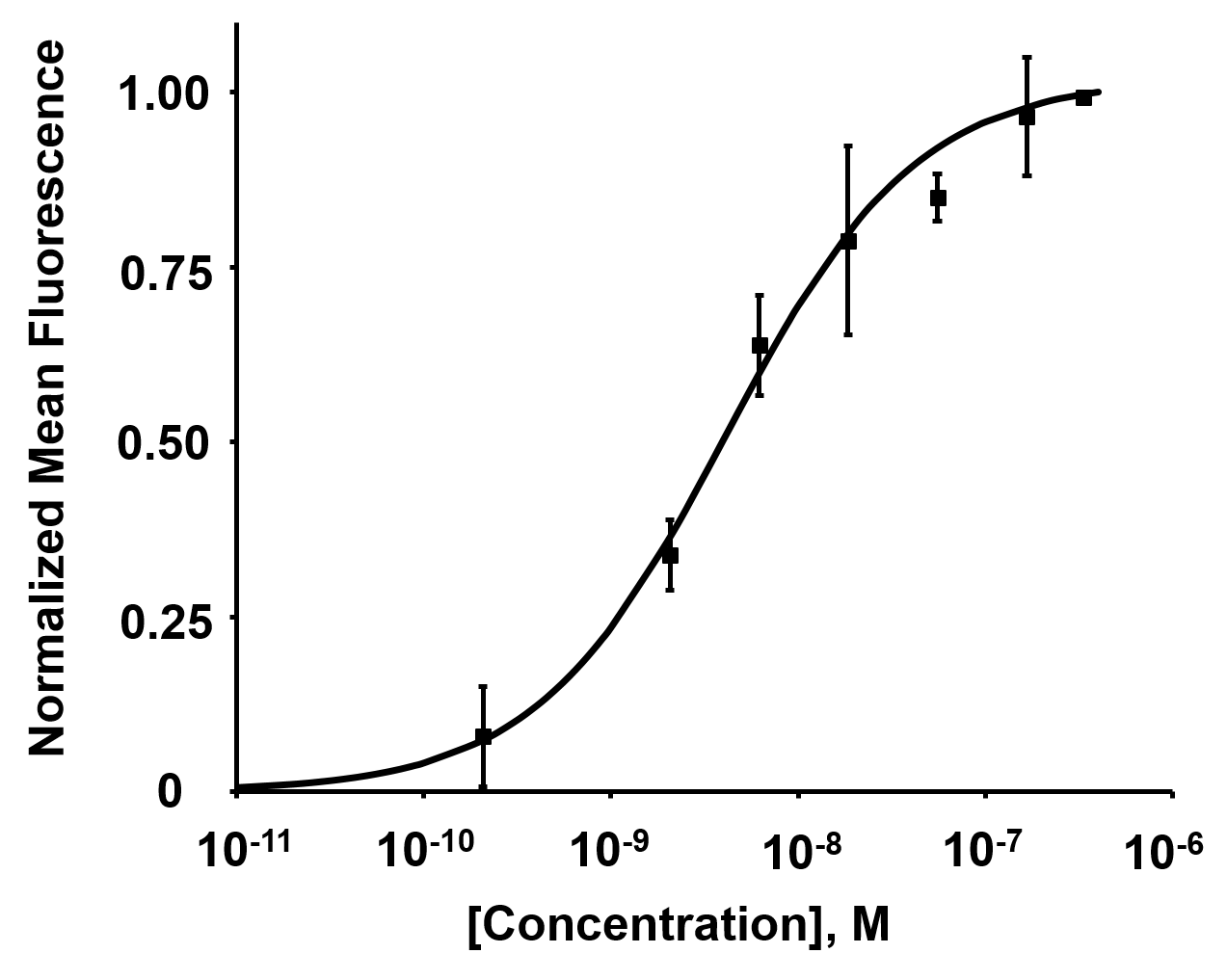


**Supporting Information Figure 4.** Binding assay with scVEGFwt on HUVECs. The K_d_ value was determined to be 3.8±0.5 nM.


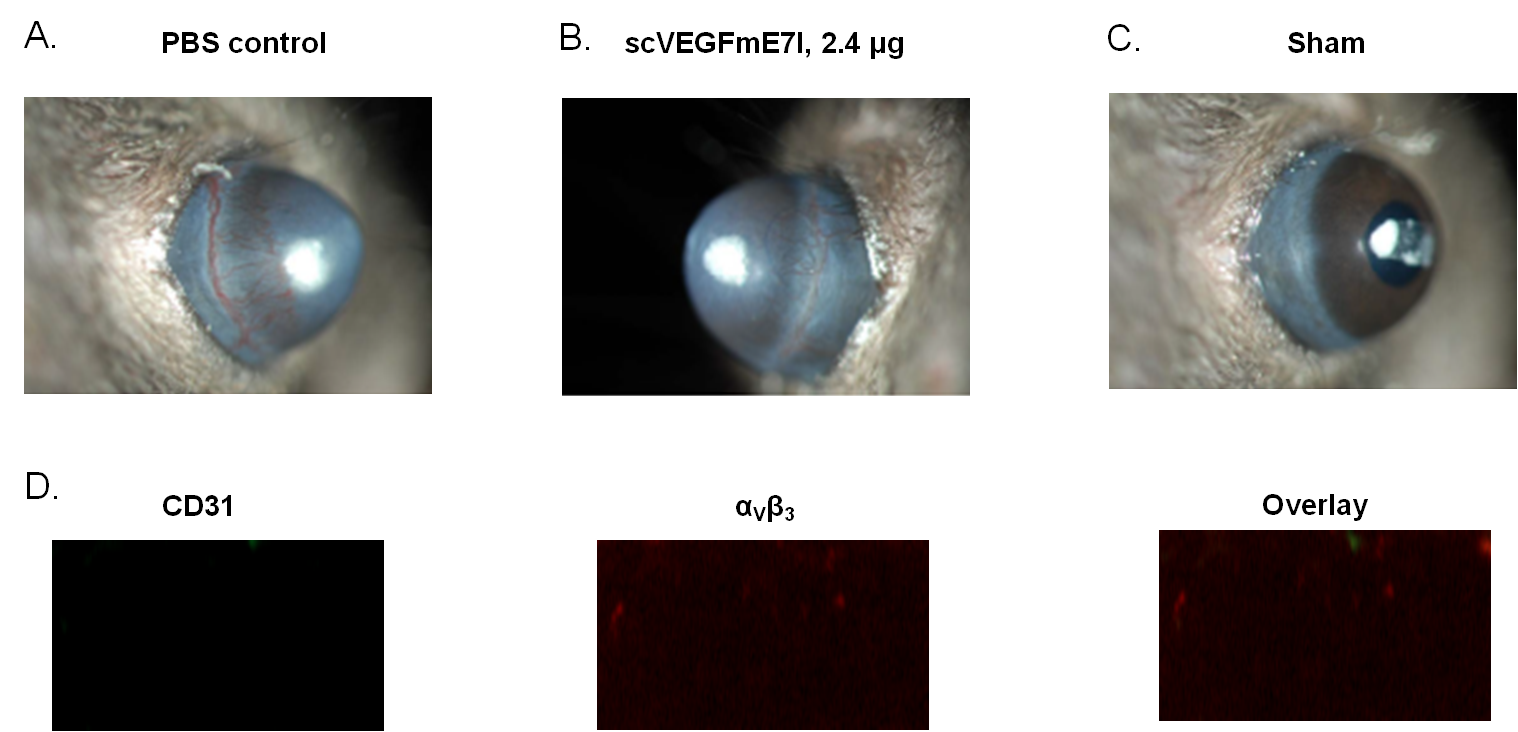


**Supporting Information Figure 5.** *In vivo* model for corneal neovascularization. (A) Representative external images of the eye from Day 6 of the model prior to euthanasia. Eyes from mice were evaluated daily, and those treated with the highest dose of scVEGFmE7I (panel B) did not show observable differences relative to the PBS control mice (panel A) for local toxicity (redness, inflammation, discharge). Marked inhibition of neovascularization is also visible and the quantification is reported in Figure 6A. The overexposed region in the image of the mouse eye originates from the implanted pellet. (C) Mouse eyes from the sham pellet control group (no growth factor) did not show any neovascularization. This confirms that angiogenesis observed in the other groups is induced only by the presence of growth factor in the pellet and not by the surgery performed to implant the pellet. (D) Immunofluorescent staining to visualize expression of α_v_β_3_ integrin in tissue isolated from mice from the sham pellet control group. CD31 was used as a marker for vasculature (endothelial cells). Two features are noteworthy. First, extra-vascular staining for α_v_β_3_ integrin that was observed in the PBS/growth factor control group (Figure 6) is absent in the sham surgery group, confirming that the presence of α_v_β_3_ integrin is associated with angiogenesis. Second, no staining for vasculature (CD31) is visible, consistent with the lack of observable angiogenesis in panel C.

**Supporting Information Table 1.** Sequences of clones isolated from scVEGFmut affinity maturation libraries. Residue numbers correspond to VEGF_121_. Mutations common to multiple unique clones are indicated in blue and other mutations are indicated in black. Residues identical to the parent scVEGFmut are in red. Specific mutations that block binding at one pole (chain 1 F17A, E64A; chain 2 I46A, I83A) are highlighted in yellow in the scVEGFmut sequence and were retained in all 14 clones sequenced.

13 82

mut EVVKAMDVYQ RSYCHPIETL VDIFQEYPDE IEYIFKPSCV PLMRCGGCCN DAGLECVPTE ESNITMQIMR

D EVVKAMDVYQ RSYCHPIETL VDIFQEYPDE IEYIFKPSCV PLMRCGGCCN DAGLECVPTE ESNITMQIMR

G EVVKAMDVYQ RSYCHPIETL VDIFQEYPDE IEYIFKPSCV PLMRCGGCCN DAGLECVPTE ESNITMQIMR

J EIVKARDVYQ RSYCHPIETL VDILQEYPDE IEYIFKPSCV PLMRCGGCCN DAGLECVPTE ESNITMQIMR

A EVAKAMDVYQ KSYCHPIETL VDILQEYPDE IGYIFKPSCV PLMRCGGCCN DAGLECVPTE ESNITMQIMR

E EVVKAMDVYQ RSYCHPIETL VDILQEYPDE IGYIFKPSCV PLMRCGGCCN GAGLECVPTE ESNITMQIMR

C EAVKAMDVYQ RSYCHPIETL VDIFQEYPDE IEYIFKPSCV PLMRCGGCCN DAGLECVPTE ESNITMQIMR

H EVVKAMGVYQ RSYCHPIETL VDISQEYPDE IEYIFKPSCV PLMRCGGCCN DAGLECVPTE ESNITMQIMR

M EVVKAMDVYR RSYCHPVETS VDILQEYPDE IEYIFKPSCV PLMRCGGCCN DAGLECVPTE ESNTTMQIMR

B EVAKAMDVYQ RSYCHPIETL VDILQEYPDE IGYIFKPSCV PLMRCGGCCN DAGLECVPTE ESNITMQIMR

83 linker 13 41

mut IKPHQGQHIG EMSFLQHNKC ECRPKKD GSTSGSGKSSEGKG EVVKFMDVY QRSYCHPIET LVDIFQEYPD

D IKPYQGHHIG EMSFLQHNKC ECRPKKD GSTPGSGKSSEGKG EVVKLMDVY QRSYCHPIET LVDIFQEYPD

G IKPYRGHHIG EMSFLQHNKC ECRPKKD GSTSGSGKSSEGKG EVVKFMDVY QRSYCHPIET LVDIFQEYPD

J IKPYQGHHIG EMSFLQHNKC ECRPKKD GSTSGSSKSSEGKG EVVKFMDVY QRSYCHPIET LVDIFQEYPD

A IKPYQGQHIG EMSFLQHNKC ECRPKKD GSTSGSGKSSEGKG EVVKFMDVY QRSYCHPIET LVDIFQEYPD

E IKPHRGQHIG EMSFLQHNKC ECRPKKD GSTSGSGKSSEGKG EVVRFMDVY QRSYCHPIET LVDIFQEYPN

C IKPHRGQHIG EMSFLQHNKC ECRPKKD GSTSGSGKSSGGKG EVVKFMDVY QRSYCHPIET LVDVFQEYPD

H IKPHQGHRIG EMSFLQHDKC ECRPKKD GSTSGSGKSSEGKG EVVRFMDVY QRSYCHPIET LVDIFQEYPD

M IKPYRGQHIG EMSFLQHNKC ECRPKKD GSTSGSGKSSEGKG EVVKFMDVY QRSYCHPIET LVDIFQEYPD

B IKPHQGHRIG EMSFLQHDKC ECRPKKD GSTSGSGKSSEGKG EVVKFMDVY QRSYCHPIET LVDIFQEYPD

42 109

mut EIEYAFKPSC VPLMRCGGCC NDEGLECVPT EESNITMQIM RAKPHQGQHI GEMSFLQHNK CECRPKKD

D EIEYAFKPSC VPLMRCGGCC NNEGLECVPT EESNITMQIM RAKPHQGQHV GEMSFLQHNE CECRPKKD

G EIEHAFKPSC VPLMRCGGCC NNEGLECVPT EESNITMQIM RAKPHQGQHI GEMSFLQHNK CECRPKKD

J EIEYAFKPSC VPLMRCGGCC NNEGLECVPT EESNITMQIM RAKPHQGQHT GEMSFLQHNK CECRPKKD

A KIEYAFKPSC VPLMRCGGCC NNEGLECVPT EESNITMQIT RAKPHQGQHI GEMSFLQHNK CECRPKKD

E EIEYAFKPSC VPLMRCGGCC NNEGLECVPT EESNITMQIM RAKPHQGQHI GEMSFLQHNK CECRPKKD

C EIEYASEPSC VPLMRCGGCC NHEGLECVPT EESNITMQIM RAKPHQGQHI GEMSFLQHNK CECRPKKD

H EIEYAFKPSC VPLMRCGGCC NNEGLECVPT EESNITMQIV RAKPHQGQHI GEMSFLQHNK CECRPKKD

M EIEYAFKPSC VSLMRCGGCC NNEGLECVPT EESNITVQIM GAKPHQGQHI GEMSFLQHNK CECRPKKD

B EIEYAFKLPC VPLMRCSGYC NNEGLECVPT EESNITMQIM RAKPHQGQHI GEMSFLQHNK CECRPKKD

**Supporting Information Table 2.** Variants isolated from scVEGF affinity maturation libraries for further characterization. Mutations listed refer to positions in VEGF_121_ numbering scheme, and are in addition to the mutations that were previously introduced to create an antagonist by blocking VEGFR2 binding at one pole of the ligand (chain 1 F17A, E64A; chain 2 I46A, I83A). Red text: Mutations in scVEGFmA and mJ are positioned on this disrupted pole and while they might be inert, they could potentially revert the ligand back to a bivalent VEGFR2 binder.

**Clone** **Chain 1 mutations** **Chain 2 mutations**

scVEGFmA V15A, R23K, F36L, E44G, H86Y E42K, D63N, M81T

scVEGFmE F36L, E44G, D63G, Q87R K16R, D41N, D63N

scVEGFmJ V14I, M18R, F36L, H86Y, Q89H D63N, I91T

Note: scVEGFmJ also has a mutation in the 14-amino acid linker that connects chain 1 and chain 2 at the 7^th^ residue (G7S).
